# Losartan Treatment Could Improve the Outcome of TBI Mice

**DOI:** 10.3389/fneur.2020.00992

**Published:** 2020-10-15

**Authors:** Jianhua Xiong, Yalong Gao, Xiaotian Li, Kai Li, Qifeng Li, Jun Shen, Zhenying Han, Jianning Zhang

**Affiliations:** Department of Neurosurgery, Tianjin Medical University General Hospital, Tianjin, China

**Keywords:** traumatic brain injury, blood brain barrier, endoplasmic reticulum stress, neuroinflammation, angiotensin II type 1 receptors blockage, tight junction proteins, losartan

## Abstract

Traumatic brain injury frequently leads to serious mortality and physical disability, yet effective treatments remains insufficient. TBI always leads to a series of secondary brain injuries including neuronal apoptosis, continuous inflammation, endoplasmic reticulum stress, and disruption of the blood-brain barrier. Sartans that block angiotensin II type 1 receptors are strongly neuroprotective, neurorestorative and anti-inflammatory. However, whether losartan, a FDA-approved and widely used drug for regulating blood pressure, is beneficial for improving the prognosis of TBI need more evidence. Through a controlled cortical impact injury mice model, we confirmed that losartan treatment could ameliorate CCI-induced secondary brain injury. We found that losartan treatment decreased brain lesion volume, neuronal apoptosis and ER stress protein ATF4 and eIF2α. Moreover, our results showed that losartan also improved neurological and motor function. It is worth pointing out that losartan increased the expression of tight junction proteins ZO-1 and alleviated brain edema and blood brain barrier leakage. Additionally, losartan inhibited pro-inflammatory factor TNF-α and improve anti-inflammatory factor IL-10. Taken together, our data demonstrated that losartan could improve the prognosis of TBI and may be a promising therapeutic method for mitigating TBI.

## Introduction

Because of its high mortality and disability, traumatic brain injury (TBI) is a serious worldwide public health and socioeconomic problem (1, 2). Although there are limited high-quality epidemiological data, ~5.3 million people in the USA suffer from a TBI-related disability (3). However, there are currently no effective drugs for treatment of TBI. The pathogenesis of TBI is complicated and includes primary and secondary injury. Primary injury is characterized by immediate mechanical stress and loss of brain tissue after trauma (4). Secondary injury involves intricate cellular and biochemical pathological events, including oxidative stress, neuroinflammation, blood–brain barrier (BBB) damage, endoplasmic reticulum (ER) stress, brain edema, and neuronal apoptosis, which can occur within minutes after TBI and can last for hours to days to months (5–8).

Excessive Angiotensin II (AngII) increases reactive oxygen species through AngII type 1 receptor (AT1R)-mediated signaling in various disorders, including hypertension, stroke, and coronary heart disease (9–12). Overactivation of the AT1R was reported to be closely related to secondary damage after TBI (13–15). Moreover, AngII-mediated oxidative signaling can be blocked by AT1R antagonists (16, 17). For example, Villapol et al. reported that candesartan and telmisartan, which can both block AT1R and activate peroxisome proliferator-activated receptor-γ (PPARγ), have therapeutic potential in TBI (18). In recent years, studies have found that the neuroprotective effects of AngII receptor blocker (ARB) drugs include modulation of microglial activation states (19), alleviating endoplasmic reticulum (20), inhibition of neuronal apoptosis (21) and inhibition of overproduction of inflammatory factors (22) and so on. Related mechanisms such as downregulation of the TLR2 signaling pathway (23), PPARγ activation (18), activating Wnt/β-catenin signaling (24) and so on. Comparison of the protective effects of different sartans on TBI mice can be found in Table 1.

**Table 1:** Comparison of the protective effects of sartans on TBI mice.

**Sartan species**	**Behavioral outcomes**	**BBB improvement**	**Neuroprotective effect**	**Involved mechanisms**	**Reference**
Candesartan	Ameliorated cognitive impairment	No mention	Positive	AT1R blocking and PPARγ activating	(18)
Telmisartan	No effect	No mention	Positive	AT1R blocking and PPARγ activating	(18)
Candesartan	Positive	Positive	Positive	No mention	(25)
Candesartan	Positive	No mention	Positive	No mention	(15)
Losartan	No mention	Positive	Positive	TGF-β signaling blocking	(26)
Candesartan	Positive	No mention	Positive	AT1R blocking and PPARγ activating	(14)

Furthermore, there is increasing evidence that AT1R antagonists are neuroprotective and anti-inflammatory (27). Nevertheless, few studies have examined the use of losartan as a therapeutic agent for reducing neuroinflammation, edema, and lesion volume following TBI.

Losartan is an FDA-approved drug used to treat hypertension (28), and acts as an AT1R antagonist to selectively block AngII from binding to the AT1R (29). Losartan was previously reported to block brain TGF-β signaling and prevent epilepsy in albumin and BBB breakdown models of epileptogenesis (26). Losartan was also neuroprotective following TBI in mice (25), and reduced hippocampal CA1 injury following cerebral ischemia/reperfusion in rats (30). Furthermore, AT1R blockade had direct neuroprotective actions in cultured microglia (16, 31). Mechanistically, the neuroprotective actions of sartans following TBI in mice were reported to involve activation of the PPARγ pathway (18). However, the therapeutic effects and mechanisms of action of losartan remain to be validated. Thus, the aim of the present study was to examine the efficacy of losartan in alleviating the pathological and neurobehavioral deficits observed following TBI in mice.

## Materials and Methods

### Experimental Groups and Drug Administration

Adult male C57BL/6 mice (8–10 weeks old, 22–25 g) were purchased from the Experimental Animal Laboratories of the Academy of Military Medical Sciences (Beijing, China). The dose of losartan (losartan potassium tablet, dissolved in 0.9% saline) was based on that previously reported (32, 33). Mice were randomly assigned to receive losartan (3 mg/kg) or vehicle (0.09% sodium chloride solution) by oral gavage at 1 h after TBI, and then once per day. All procedures were approved by the Ethics Committee of Tianjin Medical University (Tianjin, China). All experiments were performed by investigators blinded to the study groups, which were only revealed at the end of the analyses.

### Cerebral Infarction Assay

Brain infarction volume was measured using triphenyltetrazolium chloride (TTC) staining on brain tissues collected at 72 h after TBI (34). Under deep anesthesia, mice were perfused transcardially with cold PBS. The brain tissue was then obtained, incubated immediately for 15 min at −20°C. Afterwards, the frozen whole brains were sliced into 1.0-mm sections using a mouse brain slicer (Zivic Instruments, Pittsburgh, PA, USA). The brain slices were incubated in 2% (w/v) TTC (Sigma-Aldrich, St. Louis, MO, USA) dissolved in PBS for 30 min at 37°C and then transferred to 5% formaldehyde solution for fixation. The infarct area was determined by measuring the regions that lacked TTC staining, which was quantified using Image J analysis, as previously described (35).

### Rotarod Test

The rotarod test was used to evaluate systemic motor function, especially coordination and balance. Mice were placed on a rotarod apparatus set at an accelerating rotational speed of 0–40 revolutions per minute, as previously reported (36). The rod's rotational speed was accelerated from 0 to 40 rpm. The time each mouse spent on the rod was recorded. Three repeat trials were performed for each animal, and the results were calculated as the average of the three trials.

### Modified Neurological Severity Score

As previously reported (36), the modified Neurological Severity Score (mNSS) was used to assess posttraumatic neurological function. The mNSS involves motor, sensory, balance beam, and reflex tests, with scores ranging from 0 (normal function) to 18 (maximal deficit). In the present study, mNSS tests were performed on days 1, 3, 5, and 7 post-TBI. mNSS assessments were performed by two observers blinded to the treatment groups.

### Moderate CCI Model

Controlled cortical impact (CCI) injury was performed as previously described (37). Moderate CCI injury was induced using an electromagnetically-driven CCI injury device (eCCI-6.3 device; Custom Design & Fabrication, USA) and a 2-mm-diameter round impact tip (impact depth 1.2 mm, speed 5.0 m/s, dwell time 150 ms). Mice in the sham group received all procedures apart from CCI.

### Western Blotting

Western blotting (WB) was performed as previously described (38). PVDF membranes were blocked with 5% skim milk for 2 h at room temperature, and then incubated with primary antibodies against caspase-3 (1:1,000; CST, USA), ZO-1 (1:1,000; Cambridge, MA, USA), β-actin (1:1,000; ZSGB-BIO, China), ATF4 (1:1,000; CST), eIF2α (1:1,000; CST), or NeuN (1:1,000; Abcam, UK) at 4°C overnight. After incubation with secondary antibodies (goat anti-mouse or anti-rabbit IgG, 1:5,000; ZSGB-BIO) at room temperature for 1 h, the blots were then processed and analyzed.

### Morris Water Maze

The Morris water maze test was used to assess the spatial learning and memory function of mice, as previously reported (39). All mice were trained for five continuous days starting at 14 days post-TBI. The number of crosses into the platform quadrant and the percentage of time spent in the platform quadrant in 60-s intervals were recorded.

### ELISA

Total protein concentration was measured using a BCA protein assay kit (Beyotime, Shanghai, China). At 3 days after TBI, the levels of interleukin-10 (IL-10) and tumor necrosis factor (TNF-α) in the injured cortex were measured using specific ELISA kits (R&D systems, USA), as per the manufacturer's instructions.

### TUNEL Assay

The TUNEL staining procedure was then performed as previously described (40). TUNEL and immunofluorescence staining of neuronal nuclei (NeuN) were combined to evaluate neuronal apoptosis. Frozen sections were prepared 3 days post-TBI and were incubated with an anti-NeuN antibody (1:500, Abcam, Cambridge, MA, USA) at 4°C overnight. After 1 h incubation with Alexa Fluor-conjugated anti-rabbit or antimouse IgG (1:500, Thermo Fisher Scientific, Waltham, MA, USA), 50 μl of TUNEL mixture was added, and the sections were incubated for 1 h at 37°C and then incubated with DAPI. A fluorescence microscope was used to determine the number of apoptotic neuronal cells around the traumatic lesion. The neuronal apoptosis ratio was recorded for statistical analysis.

### EB Permeability Assay

Evens blue (EB) dye extravasation was used to evaluate BBB permeability, as previously described (40). Briefly, mice were injected with 100 μl of 4% EB, and then perfused with phosphate buffered saline. The ipsilateral hemisphere was then dissected, weighed, and homogenized in 0.1 g/ml N, N-dimethylformamide (Sigma-Aldrich, USA), and centrifuged for 30 min at 3,000 revolutions per min. The supernatants were collected, and the EB content in the brain tissue was calculated from the OD values (at 610 nm) using a standard curve.

### Measurement of Cerebral Edema

Brain water content was measured by the wet-dry weight ratio method, as previously described (40). The percentage of brain water content was calculated as (wet weight – dry weight)/wet weight × 100%.

### HE Staining

Brain sections were fixed with paraformaldehyde and washed three times. The method of Hematoxylin-Eosin (HE) staining is carried out according to the instructions of the manufacturer (Solarbio, Beijing, China).

### Statistical Analysis

A repeated-measures statistical approach was used to effectively control the false positive rate. Data are expressed as the mean ± standard deviation. All experiments were performed in a randomized and blinded manner. SPSS statistical software (version 23.0, IBM) was used for all statistical analyses in the present study. Comparisons between two groups were analyzed by a *t*-test, while comparisons between multiple groups were analyzed by one-way ANOVA followed by the LSD and Bonferroni *post-hoc* test. The protein band intensity for the Western blots were determined using Image J software. Measurement of fluorescence was calculated by Photoshop. Differences were considered significant at *P* < 0.05.

## Results

### Losartan Treatment Reduces Brain Injury Volume After TBI

**Figure 1:**
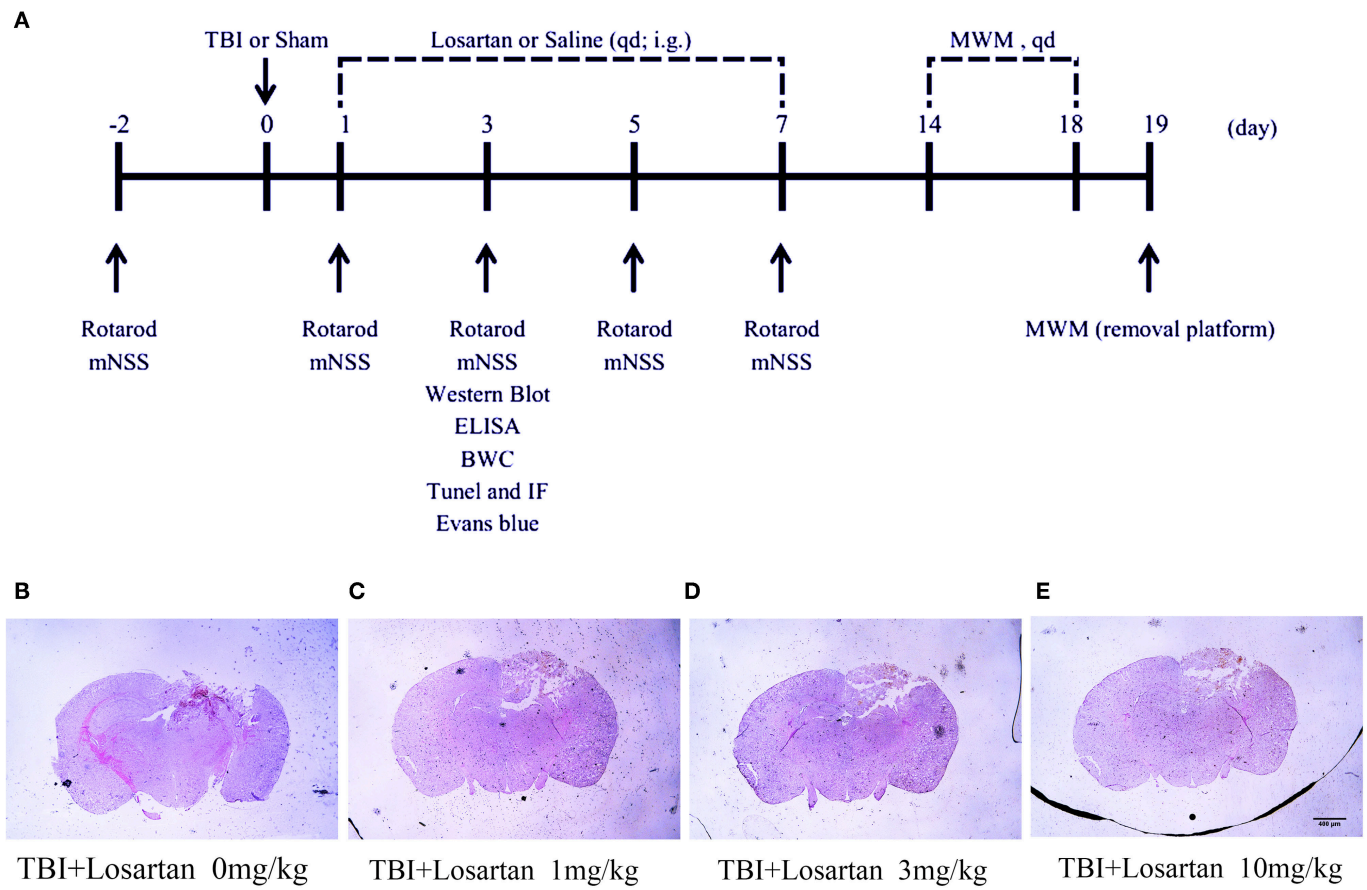
**(A)** Timeline of the animal experiment. **(B)** The volume of lesion of TBI mice after 1 day. **(C–E)** After receiving different doses of losartan (1, 3, 10 mg/kg), representative HE staining of brain tissues section at 1 day after TBI.

**Figure 2:**
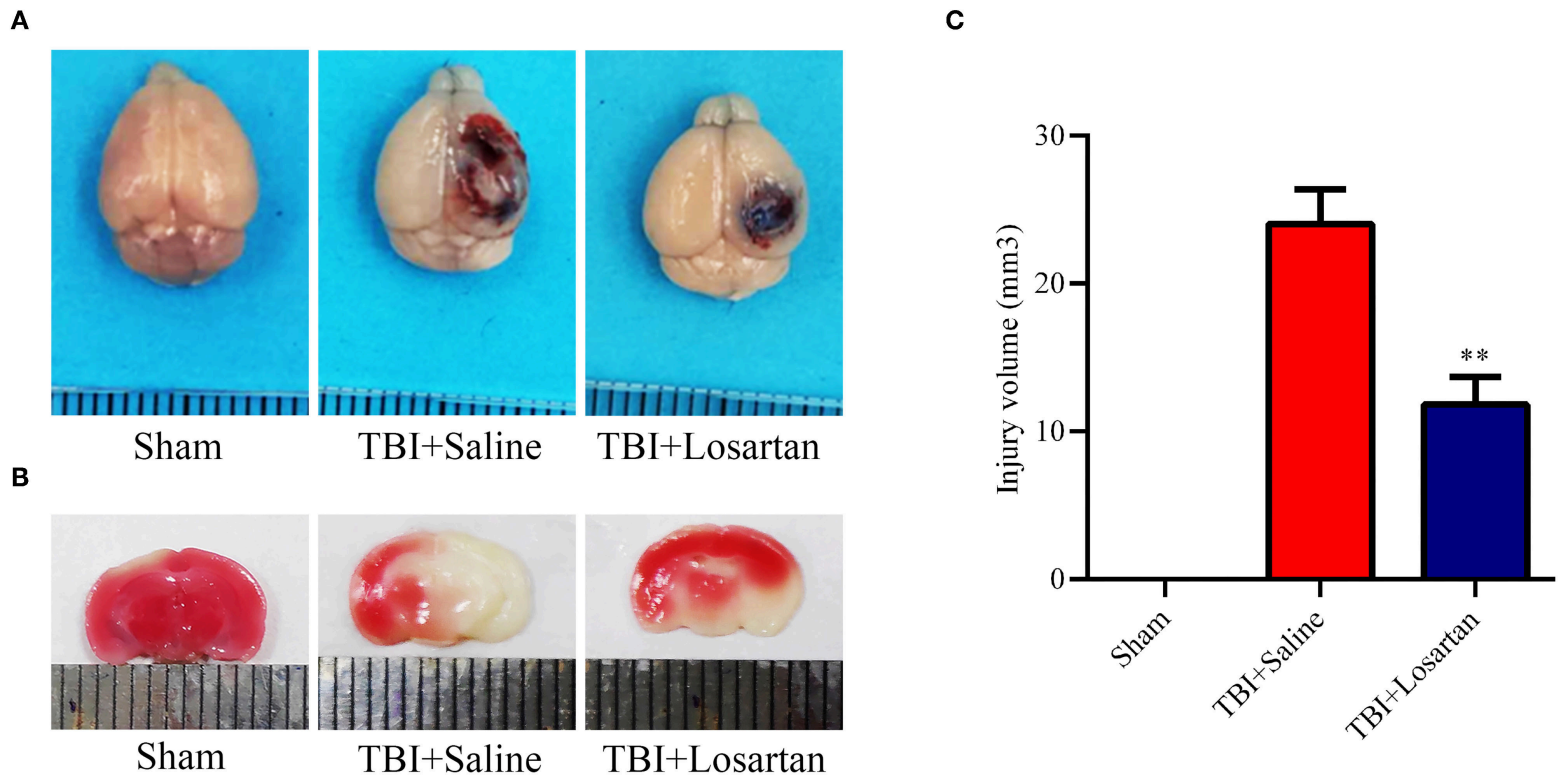
**(A)** Representative images of brain tissues 1 h after Evans blue (EB) injection at 72 h after TBI. **(B)** Representative coronal brain sections stained with TTC and the quantification of the infarct size of mice receiving losartan or vehicle on day 3 after TBI. **(C)** Effect of losartan treatment on injury volume at 72 h after TBI. All data are expressed as the mean ± SD, **p < 0.01 vs. the TBI + saline group.

The timeline of the animal experiments is presented in Figure 1A. To determine the lowest and most effective dose of losartan, we treated mice 1 h after CCI injury and sacrificed at 1 day post-injury to assess lesion volume by HE staining (Figure 1B). We determined responses to doses spanning the clinical therapeutic range for losartan (1; 3; 10 mg/kg). The lowest dose of losartan (1 mg/kg; Figure 1C) produced smaller reductions in lesion volume than the other doses (3 and 10 mg/kg). Both the middle and high doses (3 and 10 mg/kg) of losartan were equally effective in reducing the lesion volume (Figures 1D,E). We therefore proceeded using the lowest effective dose of losartan (3 mg/kg). Using EB staining, the injury volume at 3 days recovery was significant lower in the TBI + losartan group than in the TBI + saline group (Figure 2A). TTC staining confirmed that injury volume was significantly reduced in the TBI + losartan group (11.80 ± 0.94 mm^3^) compared with the TBI + saline group (24.05 ± 1.16 mm^3^; *P* < 0.01; Figures 2B,C).

### Losartan Treatment Attenuates Neurological and Motor Function Deficits After TBI

**Figure 3:**
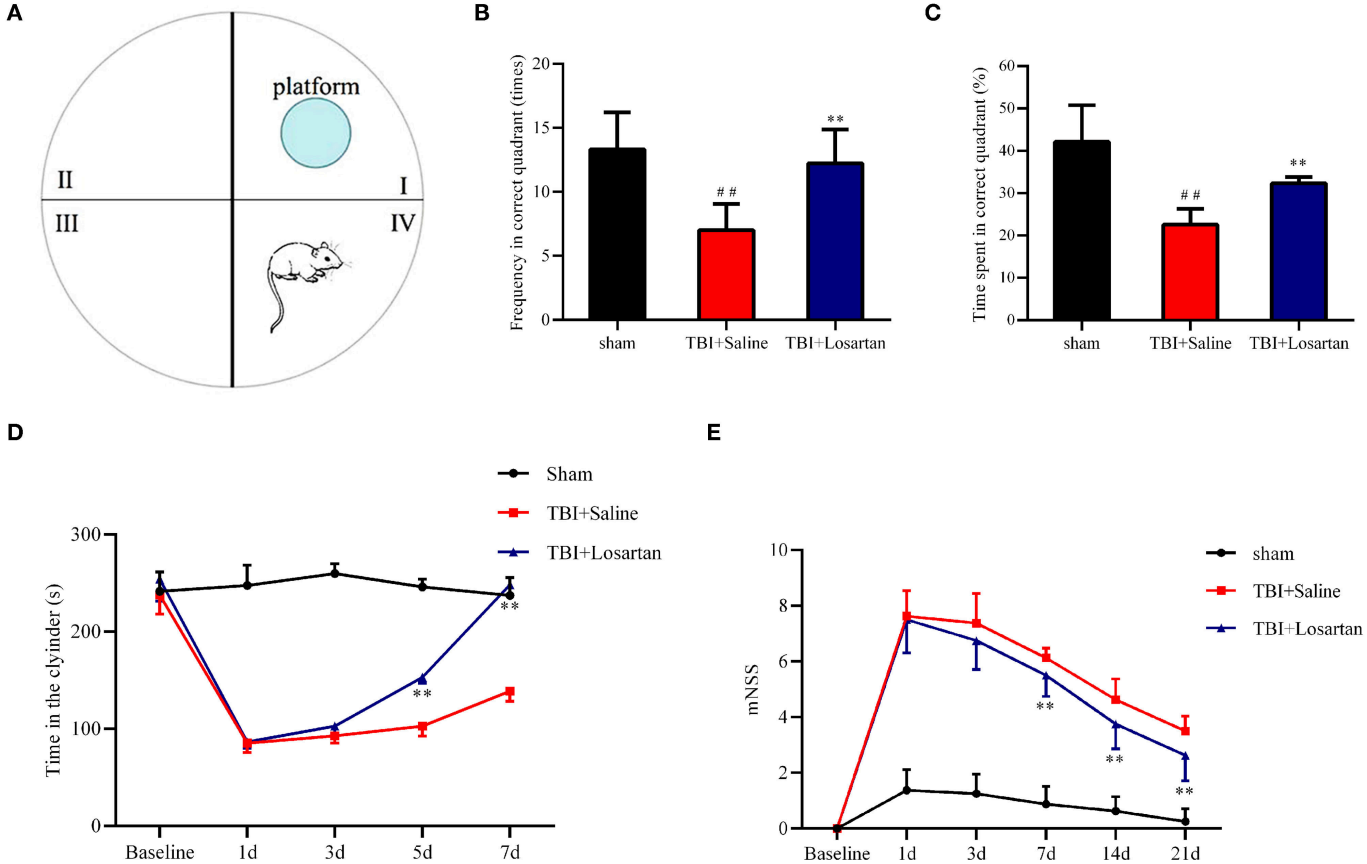
**(A)** Representative images of Morris water maze in different groups, *n* = 8/group. **(B)** Frequency through the platform quadrant of the mice between different groups. **(C)** Time percentage of the mice in the platform quadrant between different groups. **(D)** The duration time in the cylinder in the different groups at different time points. **(E)** mNSS score of mice in the different groups at different time points. All data are expressed as the mean ± SD. ^*##*^*p* < 0.01 vs. the sham group, **p < 0.01 vs. the TBI + Saline group.

The restoration of spatial memory was evaluated by the percentage of time spent in the platform quadrant and the number of crosses into the platform quadrant in 60-s intervals. The number of crosses into the platform quadrant was significantly lower in the TBI + saline group (7.44 ± 0.53) than in the TBI + losartan group (10.56 ± 0.73; *P* < 0.01) and the sham group (11.78 ± 0.62; *P* < 0.01; Figures 3A,B). Similarly, the percentage of time spent in the platform quadrant was significantly lower in the TBI + saline group (23.14 ± 1.53%) than in the TBI + losartan group (31.39 ± 1.57%; *P* < 0.01) and the sham group (41.46 ± 1.38%; *P* < 0.01; Figures 3A,C). Overall, these data suggest that losartan treatment can improve TBI-induced cognitive deficits.

Motor coordination and balance was evaluated using the rotarod test. There were no significant difference in the time spent on the cylinder between the TBI + saline group and the TBI + losartan group at 1 day (*P* = 0.837) and 3 days (*P* = 0.204) after TBI. However, the time spent on the cylinder was significantly lower in the TBI + saline group than in the TBI + losartan group at 5 d and 7 d after TBI (*P* = 0.004, and *P* = 0.002, respectively). Overall, these data suggest that losartan treatment can improve the motor dysfunction caused by TBI (Figure 3D).

The neurological function of the mice was assessed by mNSS. There was a significant increase in the mNSS after TBI. At 3 days after TBI, the neurological function of mice had gradually recovered in the TBI + losartan treatment group, while the mNSS was significantly lower in the TBI + losartan treatment group than in the TBI + saline group on 3 days (*P* = 0.001), 5 days (*P* < 0.01), and 7 days (*P* < 0.01) after TBI (Figure 3E).

### Losartan Treatment Alters Cytokine Levels in the Peri-Contusional Cortex After TBI

**Figure 4:**
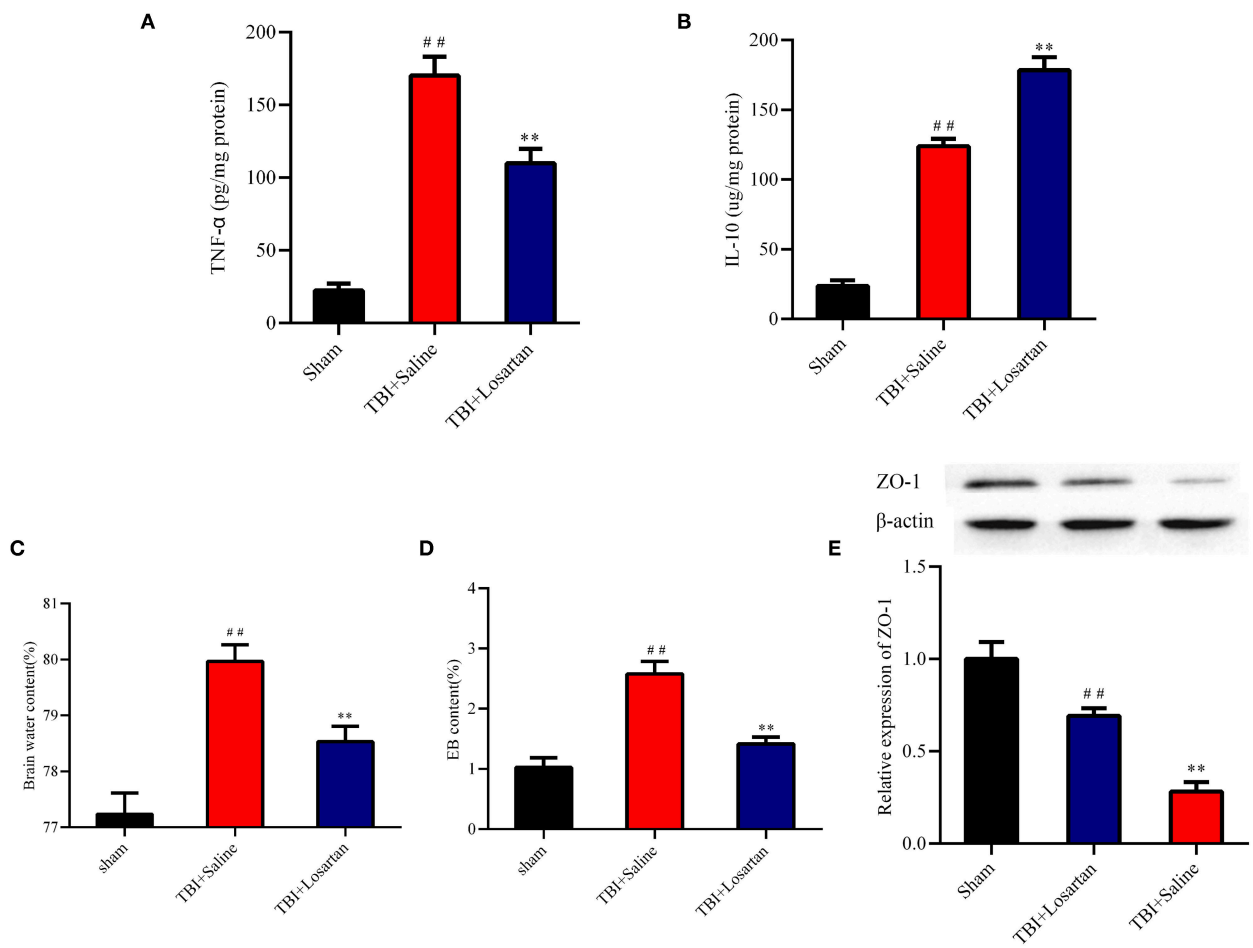
Effect of losartan treatment on inflammatory cytokines expression in peri-contusional cortex at 72 h after TBI. Compared with the TBI + saline group, losartan treatment inhibited the concentrations of pro-inflammatory TNF-α **(A)** and increased anti-inflammatory cytokines IL-10 **(B)** in brain. **(C)** Effects of losartan on the brain edema at 72 h after TBI. **(D)** Brain water content was detected at 72 h after TBI. ##*p* < 0.01, vs. sham group, **p < 0.01, vs. TBI+Saline group. **(E)** Effects of losartan on the expression level of tight junction protein 1 (ZO-1) at 72 h after TBI (*n* = 4). All the results are expressed as the mean ± SD, and *n* = 5 for each group. ^*##*^*p* < 0.01, vs. sham group, **p < 0.01, vs. TBI + Losartan group.

ELISA was used to determine the effect of losartan on anti-inflammatory (IL-10) and pro-inflammatory (TNF-α) cytokine protein expression in the peri-contusional cortex. IL-10 and TNF-α expression were both low in the sham group. TNF-α expression was significantly lower in the TBI + losartan group than in the TBI + saline group (*P* < 0.01; Figure 4A). Similarly, IL-10 expression was significantly higher in the TBI + losartan group than in the TBI + saline group (*P* < 0.01; Figure 4B).

### Losartan Treatment Decreases EB Permeability and Brain Water Content and Improves BBB Integrity After TBI

Next, we measured EB permeability and brain water content to determine the effect of losartan treatment on BBB injury following TBI. At 3 days after TBI, there was a significant increase in brain edema in the TBI +saline group (79.97 ± 0.12%) compared with the sham group (77.23 ± 0.15%; *P* < 0.01), which was significantly reduced in the TBI + losartan group (78.53% ± 0.11%; *P* < 0.01; Figure 4C). Moreover, EB permeability was significantly higher in the TBI + saline group (2.58 ± 0.21 ug/g) than in the sham group (1.03 ± 0.16 ug/g; *P* < 0.01) and the TBI + losartan group (1.41 ± 0.11 ug/g; *P* < 0.01; Figure 4D). TBI is associated with an increase in BBB permeability via downregulation of the tight junction protein ZO-1, which is an important protein that maintains the integrity of the BBB. At 3 days after TBI, there was a significant decrease in ZO-1 expression in the TBI + saline group compared with the sham group (*P* < 0.01) and the TBI + losartan group (*P* < 0.01; Figure 4E).

### Losartan Treatment Reduces ER Stress After TBI

**Figure 5:**
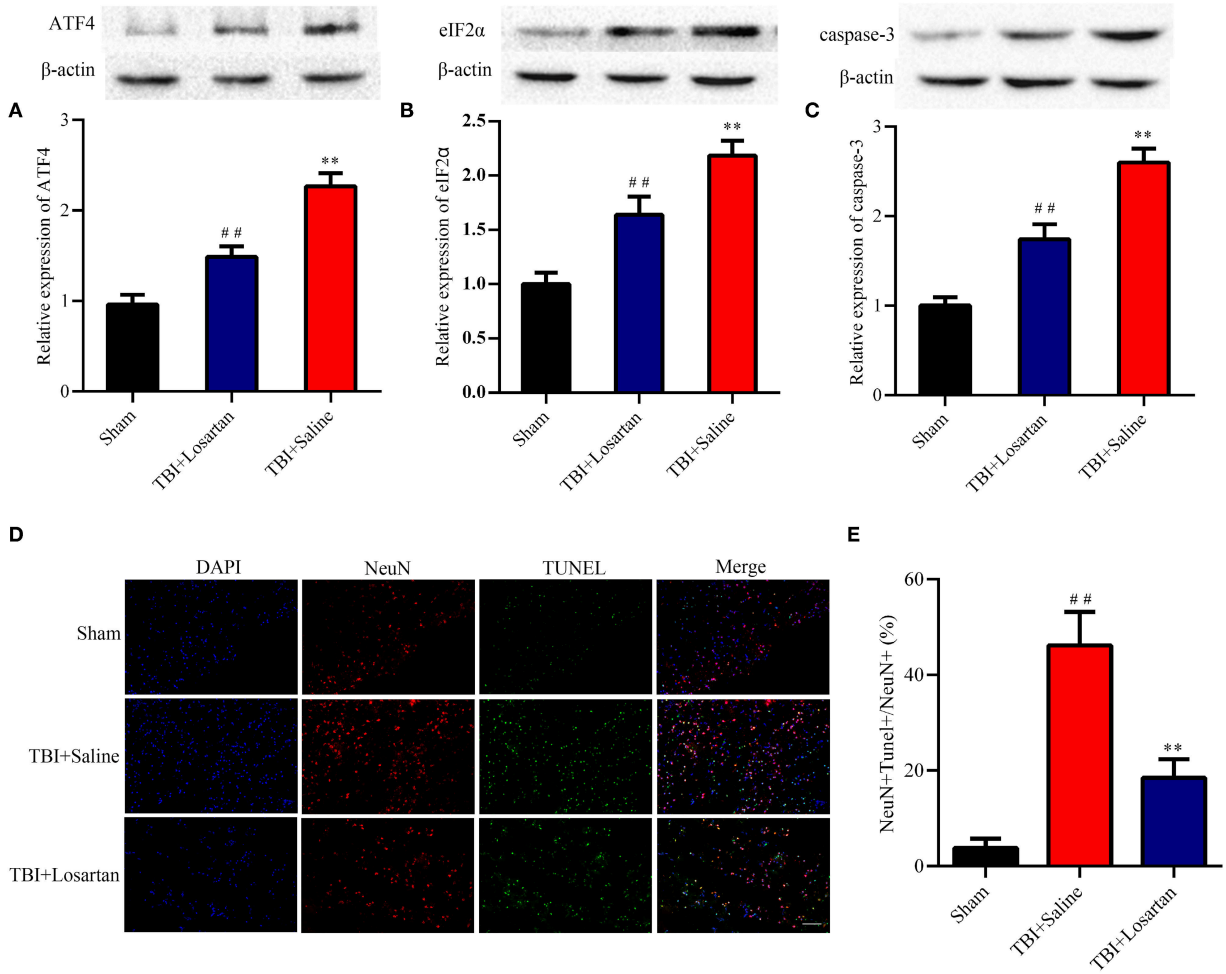
Effects of losartan on the expression level of ER stress-associated proteins, and apoptosis protein at 72 h after TBI. Representative results of ATF4 **(A)**, eIF2α **(B)**, and caspase-3 **(C)** alteration in the different groups. All the results are expressed as the mean ± SD, and n = 4 for each group. **p < 0.01 vs. sham; ^*##*^*p* < 0.01 vs. TBI+saline. **(D)** Representative fluorescence of apoptotic neurons in the cortex of peri-lesion at 3 days after TBI. Fluorescence colors: NeuN: red, terminal deoxynucleotidyl transferase-dUTP nick end labeling (TUNEL): green, and DAPI: blue. Scale bar = 400 μm. NeuN and TUNEL double stained cells represented the apoptotic neuron. **(E)** Quantification of apoptotic neurons between the different groups. ** < 0.01, vs. TBI + Saline group, ## < 0.01, vs. Sham group.

TBI was associated with an increase in ER stress markers, which peaked at 3 days after TBI according to previous research (37). In our study, treatment with losartan significantly decreased the expression of ATF4 (*P* < 0.01; Figure 5B) and eIF2α (*P* < 0.01; Figure 5A) compared with the TBI + saline group.

### Losartan Treatment Decreases Apoptosis After TBI

Caspase-3 is a key protein in the process of cellular apoptosis. By WB, we confirmed that the expression of caspase-3 was significantly higher in the TBI + saline group than in the TBI + losartan group (Figure 5C). Next, the TUNEL assay and NeuN double-staining were used to quantify neuronal cell apoptosis after TBI (Figure 5D). There was a significant increase in the percentage of TUNEL-positive neurons in the lesion area of the ipsilateral cortex in the TBI + saline group compared with the sham group (*P* < 0.01) and the TBI + losartan group (*P* < 0.01; Figure 5E).

## Discussion

AT1R are widely distributed throughout the central nervous system, and are involved with many important regulatory functions (41). It is well–established that increased AngII signaling *via* the AT1R has detrimental effects in stroke (42) and on cognitive function (43). In the present study, we assessed the potential of targeting AT1R signaling in ameliorating neurological damage after TBI. Abdul-Muneer et al. found that losartan can mitigate neuronal damage caused by AngII in a neuronal stretch-injury model *in vitro* (44). Our *in vivo* findings show that losartan treatment can also reduce brain lesion volume at 72 h after TBI, via inhibition of apoptosis in the ischemic penumbra.

The Morris water maze is a robust and reliable test of spatial learning and memory, and is strongly correlated with hippocampal synaptic plasticity and N-methyl-D-aspartate (NMDA) receptor function in rodents (39). We found that losartan treatment improved both the cognitive and motor dysfunction caused by TBI, and also improved overall neurological function. It was previously reported that the neuroprotective action of losartan after cerebral ischemia/reperfusion injury involved inhibition of the AT1/ASK1/MKK4/JNK3 pathway in the hippocampal CA1 region (30). These findings imply that losartan has both acute and long-term benefits. However, further studies are required to examine the efficacy of long-term losartan treatment.

These is accumulating evidence that TBI is associated with neuroinflammation involving microglial and astrocyte activation, and that the neuroinflammatory cascade can control the development of cerebral edema, BBB disruption, and secondary neuronal injury (45). The result that losartan treatment significantly improve the ratio of anti-inflammatory (IL-10) to pro-inflammatory (IL-6) cytokines in the brain after TBI may be one of the mechanisms of mouse recovery. Furthermore, losartan can directly act on microglia or astrocytes, and their protective benefits were verified in comparable studies (33, 46, 47). Previous studies have also confirmed that excessive AngII can activate rat neurons and microglia *in vitro*, and that losartan can block this effect (31, 44). Additionally, Benicky et al. reported direct anti-inflammatory effects of sartans in cultured rat microglia, cerebellar granule cells, and cerebral microvascular endothelial cells (16). Overall, these findings indicate potential mechanisms underlying the neuroprotective actions of losartan in the present study. Markers associated with glial cell activation including GFAP and Iba-1 are necessary to verify in order to obtain reliable evidence.

AngII plays a pivotal role in secondary injury after TBI. Overproduction of AngII following TBI can activate oxidative stress and caspase-3 pathways, which can result in cellular apoptosis (44). The BBB is composed of pericytes, astrocytes, endothelium, and tight junction proteins, and is surrounded by neurons (48, 49). After TBI, excessive oxidative stress can directly downregulate the expression of ZO-1 (50), leading to increased BBB permeability. At the same time, neuronal loss can also contribute to BBB damage. In the present study, we found that losartan treatment after TBI could block these upstream processes, and thus improve BBB integrity *in vivo*.

Previous studies have reported that candesartan and telmisartan treatment can improve recovery from TBI via a combination of PPARγ agonist activity and AT1R blockade (18), which may represent a mechanism of action for losartan. We also found that losartan treatment suppressed ER stress after TBI. ER stress can disturb the inflammatory response following TBI via inhibition of the nuclear factor kappa-B (NF-κB) signaling pathway (51), and lead to neuronal death through ATF4 (52). In support, the neuroprotective actions of candesartan were reported to involve inhibition of ER stress in a rotenone model of Parkinson's disease in rats (53), potentially via inhibition of the ATF4-CHOP-Puma pathway. Thus, the therapeutic effect of losartan on TBI in the present study may relate to suppression of ER stress, although further studies are required to determine the specific signaling mechanisms.

Losartan was originally used for treatment of hypotension, although it was also reported to have cardiovascular (54, 55) and renal protective effects (56) unrelated to blood pressure. Cerebrovascular hemodynamic changes are often observed following TBI, which are associated with poor prognosis. So, does losartan affect blood pressure? Our study found that the blood pressure of TBI mice taking losartan was a little low, but there was no statistical difference between the sham group and the TBI group (data not shown). Previous research has shown that the dose of losartan (3 mg/kg daily) used in the present study was low, without any significant effects on cerebral perfusion (57). Moreover, other studies confirmed that greater doses of losartan will not affect blood pressure in mice, which is consistent with our results (58). Thus, our neuroprotective actions of low-dose losartan are unlikely related to effects on blood pressure.

## Conclusion

Our study provides new evidence that losartan exerts short- and long-term protection against secondary brain injury caused by TBI. Specifically, losartan treatment reduced neuronal apoptosis and ER stress, enhanced BBB integrity, and improved cognitive and motor function, as well as overall neurological function. These findings suggest that losartan is a potential candidate for treatment of TBI. Future studies are required to determine the specific mechanisms of action of losartan in secondary brain injury, including the signaling pathways and effects on different cell types, including astrocytes, microglia, and endothelial cells.

## Data Availability Statement

The raw data supporting the conclusions of this article will be made available by the authors, without undue reservation.

## Ethics Statement

The animal study was reviewed and approved by Institutional Ethical Review Committee of The General Hospital of Tianjin Medical University.

## Author Contributions

JZ: conception and design of the manuscript. JX: drafting of the manuscript. YG, XL, and KL: critical revision of the manuscript for important intellectual content. XL and KL: manuscript supervision. JX, YG, QL, and ZH: final approval of the revised manuscript. All authors contributed to the article and approved the submitted version.

## Conflict of Interest

The authors declare that the research was conducted in the absence of any commercial or financial relationships that could be construed as a potential conflict of interest.
